# Youth-centered Recommendations to Address Social Stigma and Discrimination Against Unhoused Youth: An Integrative Literature Review

**DOI:** 10.1177/10598405231214061

**Published:** 2023-11-22

**Authors:** Huy Le, Lynn Rew

**Affiliations:** 1 The University of Texas at Austin School of Nursing, Honors Program, TX, USA; 2 Denton & Louise Cooley and Family Centennial Professor in Nursing, 12330 The University of Texas at Austin, TX, USA; 3 The University of Texas at Austin School of Nursing, Austin, TX, USA

**Keywords:** unhoused youth, homelessness, stigma, discrimination, socio-ecological, intersectionality, housing, school, policy, services, integrative literature review, school nursing, school nurses

## Abstract

Youth between ages 13 and 25 who experience homelessness face numerous barriers to excellent health, including social stigma and discrimination. Applying socio-ecological model and intersectionality theory, an integrative literature review was conducted. Peer-reviewed studies (*N *= 29) representing 808,296 participants extracted from four databases (CINAHL, MEDLINE, PsychINFO, SocINDEX) were analyzed. The studies included sources of discrimination and stigma from interpersonal interactions with support services staff upwards to policy and systemic levels with housing and justice systems. Health outcomes include poorer physical and behavioral health status from increased likeliness of denied access to support services, prolonged time spent being homeless, and higher incidences of experiencing violence. School nursing has power to push for recommended changes and support unhoused youth towards excellent health. Proposed changes include adapting Housing First framework, engaging with unhoused youth in program planning, policy writing, and public education that address the causes of poverty.

Youth progress through transformative experiences during the period of 13–25 years of age. In a span of 12 years, youth can have life-lasting positive health outcomes if their needs are adequately met; however, youth who experience homelessness (YEH) do not have similar opportunities to fulfill basic needs. Federal definitions of YEH vary by programs. McKinney-Vento Homeless Assistance Act defines YEH as people who “lack fixed, regular, and adequate nighttime residence,” meaning loss of housing, living in temporary residences, public spaces, transportation, suboptimal housing, and migrant children (The McKinney-Vento Homeless Assistance Act, 2009, pg. 1). YEH also include street youth, runaway youth, throwaway youth, and systems-involved youth (Hammer et al., 2002; youth.gov, n.d.). Systems-involved youth are youth who have been part of the juvenile justice system, child welfare system, or both (also known as crossover youth).

Nearly 4.5 million youth experience homelessness each year, more than the population of the city of Los Angeles (Morton et al., 2017). Roughly 50% of YEH reported that it was their first time being unhoused (Morton et al., 2017). There are several known precipitating factors of youth homelessness. Domestic violence, interpersonal conflicts, lack of affordable housing and household financial insecurity, ageing out of the foster care system, history of incarceration, untreated mental health conditions, and inadequate social network support or rejection are major causes of homelessness (Aratani, 2009; Kelly, 2020). Youth may have additional social vulnerabilities to homelessness through oppression based on race, ethnicity, sex, gender, ability, and age. Youth who are Lesbian, Gay, Bisexual, Transgender, and Queer (LGBTQ+), females, Black, non-White Hispanic, 18–25 years old, and/or have disabilities experience disproportionate rates of homelessness (Morton et al., 2018). Therefore, solutions to address youth homelessness call for a socially committed, interdisciplinary, and equitable approach.

## Nursing and Homelessness

YEH encounter various negative social determinants of health (SDOH), such as food insecurity, lack of safe shelter and hygiene facilities, unstable access to electricity and internet, disconnection from supportive social networks, changing education and lack thereof, limited employment opportunities, and insufficient healthcare access (Substance Abuse and Mental Health Services Administration [SAMHSA], 2019). YEH have a higher risk of exposure to adverse childhood experiences (ACEs) than housed youth (Barnes et al., 2021). ACEs can lead to more vulnerability to chronic diseases, worse mental health outcomes (Gewirtz O’Brien et al., 2020), being victims of interpersonal violence (Tyler & Schmitz, 2018), and increased risk of contracting communicable diseases (Strashun et al., 2020). Caring for youth experiencing homelessness calls for the combined disciplines of public health and nursing. The Public Health Nursing model emphasizes relationship-building, population-based, holistic, health-promoting factors, systems-based interventions, and commitment to social justice (Keller et al., 2011). Nurses act as mediators to improve health and advocate for YEH. Nurses provide essential healthcare to unhoused youth in school settings, hospitals, community clinics, and outreach services (Weber 2019). Nurses also have the capacity to advocate for primary prevention of homelessness, such as identifying causes of poor health outcomes, building community, collecting epidemiological data, and writing policies through community-based partnerships (Weber 2019). Therefore, the nursing profession is called to identify how youth homelessness is perpetuated within society, participate in drafting goals for lasting positive health outcomes, and advocate for social interventions that place health promotion at the center.

## Intersectionality in Stigma and Discrimination

Social stigma and discrimination contribute to the perpetuation of youth homelessness. Social stigma represents the gap between sets of expectations socially assigned to groups of individuals to categorize people for effective interaction and the complete representation of each person who may share backgrounds (Goffman, 1986). Unhoused youth experience frequent social stigma, preventing them from accessing support services due to an unwelcoming environment. Through society-driven stereotypes and judgment, unhoused youth are assigned labels and viewed as outcasts. Discrimination manifests as actions derived from stigma that intentionally limits and disadvantages certain groups of people, rooted in differences in power and access to resources (Kohler-Hausmann, 2011). Discrimination against YEH denies access to appropriate shelters and high-quality healthcare, criminalizes disproportionately, and prevents youth from exiting homelessness. Based on the frequency and pervasive nature of social stigma and discrimination against YEH, it is imperative to map how, where, and who drives these consequential inequities.

Unhoused youth also experience other forms of identity-based mistreatments. Intersectionality theory allows a fuller examination of how stigma and discrimination interact across identities. In 1989, Dr. Kimberlé Crenshaw wrote a paper originating the term “intersectionality.” Crenshaw argued that discrimination against Black women has to consider structures against both being Black and being a woman (1989). By acknowledging the dynamics of both racism and sexism, oppression and how to address it can be seen as a more complete phenomenon. In this paper, intersectionality is used to include how racism, sexism, ableism, and other forms of structural oppression further compound challenges experienced by unhoused youth.

## School Nursing

Youth between the ages of 13 and 25 years spend a great deal of time in school. School is an essential part of the socialization of all children in the United States. It is also a place where children and youth begin to experience the discrimination and stigma that accompany any attribute that makes them stand out as different from the majority. School nursing is a vital component of the Public Health discipline that is committed to promoting the health and well-being of children and youth (Kindi et al., 2021). The American Academy of Pediatrics (2016) recommends that every school has at least one registered nurse, but there is no current data available from the Department of Education to determine if this recommendation is being met (National Center for Education Statistics, 2020).

School nurses often witness how the SDOH affect the wellbeing of students, but school nurses may lack sufficient tools to support the unique needs of unhoused students and address structural causes of homelessness. SDOH are referred to as social factors, such as housing, education, environment, food, transportation, safety, social networks, etc. that can influence health outcomes. In this case, school nurses must seek additional preparation through continuing and advanced education to understand drivers of social stigma and discrimination processes. Their communication with and support of all students is an important first step in modeling acceptance of all ways of being. School nurses are well-positioned as coordinators in connecting unhoused students to essential resources by building trusting relationships and collaborating with community partners (Hindman & Mincemoyer, 2014). School nurses can develop programs that promote safe learning environments and address misinformation that drives stigma and discrimination against unhoused students. They can also work with their School Board to establish policies that encourage the acceptance of all youth despite their backgrounds. It is important to recognize that school nurses are often under-resourced within their scope of practice. School administrators need to create protected times for school nurses to learn, create, and manage initiatives supporting unhoused students.

Unhoused students may experience other identity-based stigma and discrimination, such as homophobia and transphobia. A study of health professionals, including school nurses, in New Mexico showed that they lacked preparation in providing safety and support for LGBTQ+ students (Mahdi et al., 2014). Many of the school nurses in this sample were not aware of the connection between loneliness and stress experienced by this group of students and their risk for suicide. Moreover, they felt ill-prepared to address the health disparities and health risk behaviors of these students. In another study of LGBTQ+ students and school health professionals in Massachusetts, researchers found that these students had many unmet health needs, including being harassed and experiencing sexual violence at school (Sava et al., 2021). These findings highlight the need for school nurses to be equipped with knowledge on intersectionality and structural competency to address social stigma and discrimination among unhoused students. This integrative literature review aims to raise awareness of complex social dynamics experienced by unhoused youth for school nurses so that holistic care can be achieved.

## Theoretical Frameworks

We thus propose to answer the following research questions in this integrative literature review:

1. How do unhoused youth experience social stigma and discrimination?
2. What are YEH health outcomes associated with social stigma and discrimination?
3. How can healthcare and society-at-large take evidence-based suggestions to build an equitable and inclusive support system for unhoused youth?

An integrative literature review includes both quantitative and qualitative data, effectively mapping the prevalence and nature of social stigma and discrimination against YEH. A socio-ecological model was used to conceptualize how each layer of society interacts with each other in producing a negative image of unhoused youth and prevents optimal health outcomes (Kilanowski, 2017). Socio-ecologcial model is composed of internalized/individual, interpersonal, community, institutional, public policy, and society levels. Internalized/individual level is considered how one views oneself, inclusive of lived experiences. Interpersonal level depicts the quality of relationships and interactions, such as connecting with a friend. The community level can be analyzed through social public spaces, local politics, and community attitudes around geo-spatial access. Institutional-level examples are schools, hospitals, churches, employers, and service organizations. At public policy level, factors such as conditions to qualify for housing, school disciplinary policies, intensity and scope of policing, and who gets access to basic resources are studied to conceptualize how laws and ordinances shape communities, institutions, and people's behaviors. Zooming out to the society level, norms towards a certain group of people are used to maintain differential levels of power, privilege, and access, such as classism, racism, etc. Intersectionality, a critical framework showing how systems work interconnectedly to produce outcome differences, was used to understand how YEH and who manifest oppressed identities further intensifies the impact of stigma and discrimination (Crenshaw, 2019). For example, up to 30% of unhoused transgender individuals experience shelter access denial and more than 20% experience sexual assault from shelters staff or residents in comparison to cisgender unhoused individuals; transgender women are at higher percentage of shelter access denial in comparison to transgender men (34% vs. 20%) (Grant et al., 2011). Furthermore, this study further brings visibility to youth homelessness and provides actionable steps toward a future of acceptance, acknowledgement, accessibility, and availability of resources.

## Methods

The primary literature review method used is an integrative literature review, following general guidelines by Fink (2020) and recommendations by Whittemore and Knafl (2005).

### Eligibility Criteria

Inclusion criteria for studies included participants between the ages of 13–25 years, conducted in the United States, peer-reviewed, addressed the experience of homelessness, and discussed social stigma and/or discrimination. Through consultation with a university-affiliated librarian, other search terms related to homelessness were included: Unhoused, houseless, street living, unsheltered, unstable living. Additionally, other search terms related to stigma and discrimination were included: Exclusion, marginalization, attitudes, alienation, prejudice, welcome, and acceptance. Excluded studies included op-eds, commentaries, letters to editors, reviews, case reports, abstract only or lack thereof, and dissertations.

### Search and Article Selection Process

**Figure 1.**
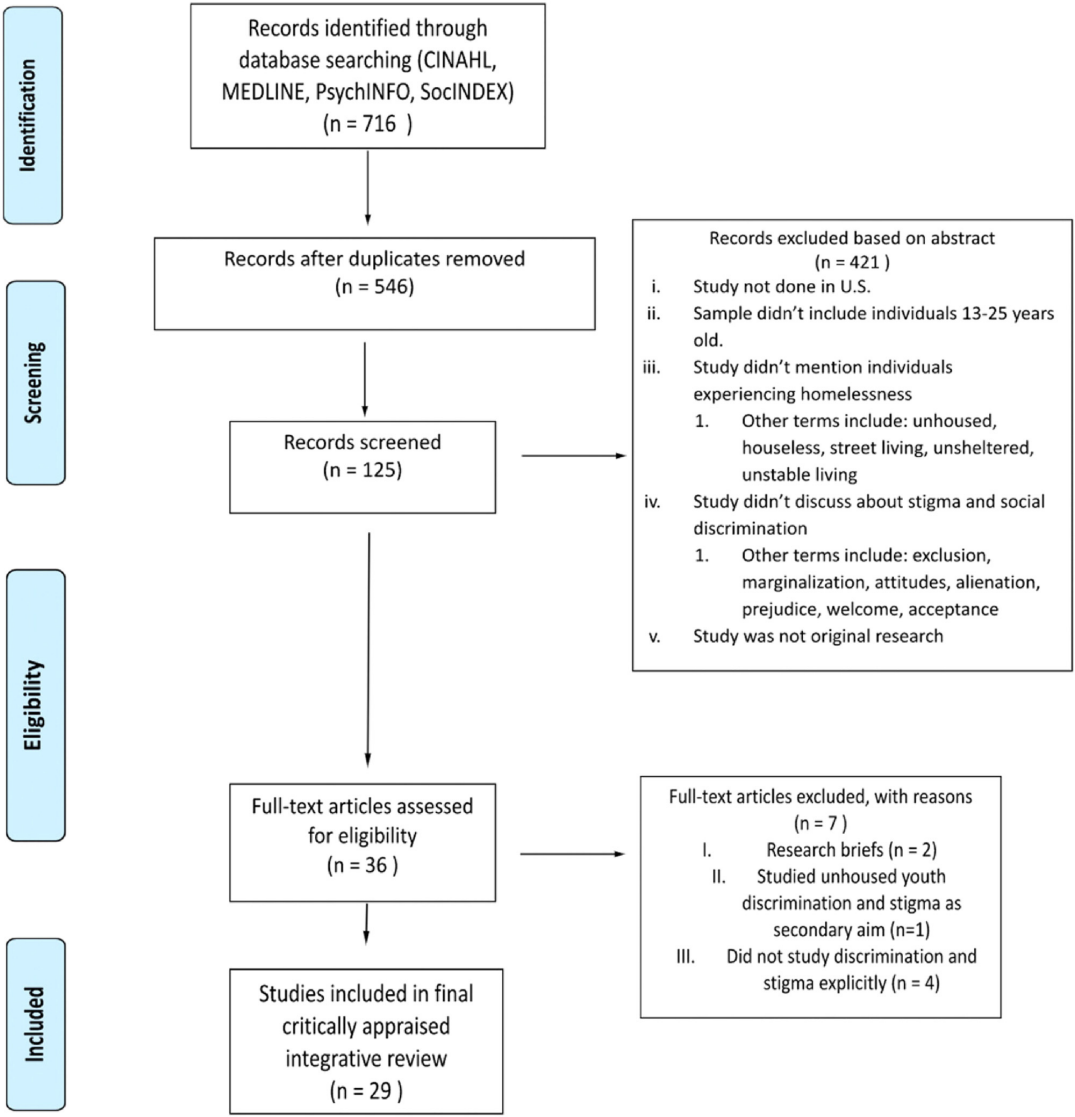
Preferred Reporting Items for Systematic Reviews and Meta-Analyses (PRISMA) diagram showing identification, screening, eligibility, and included articles.

**Figure 2.**
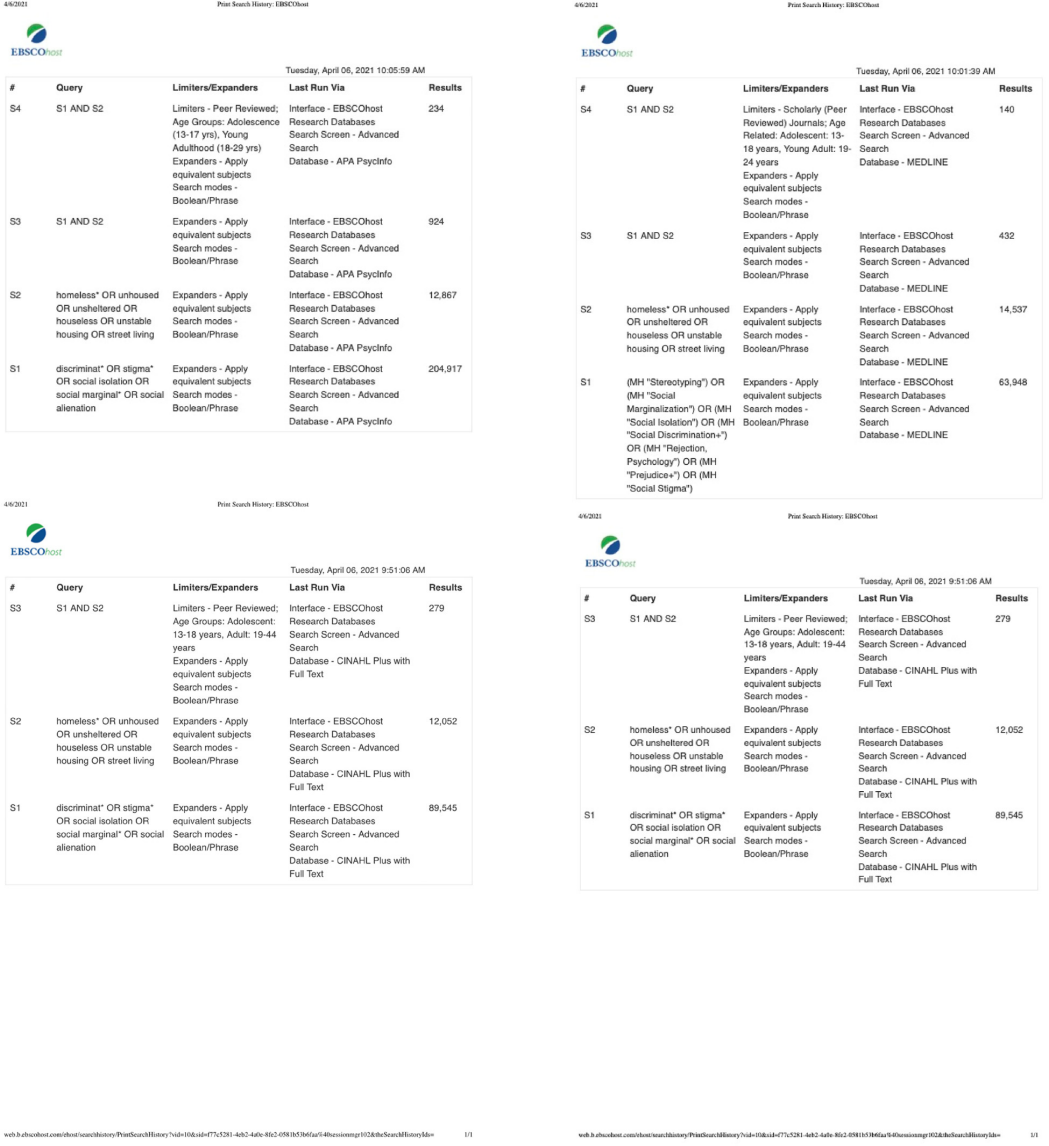
Search terms and filters used to find articles from CINAHL, MEDLINE, PsycInfo, and SocINDEX.

Through the suggestion from the librarian for high-yield studies, CINAHL, MEDLINE, PsychINFO, and SocINDEX databases were searched to find studies related to YEH and social stigma and discrimination. The resulting studies were extracted from these databases in April of 2021. There is no parameter for year selected in order to be as comprehensive as possible. A sample of 279 articles from CINAHL, 140 articles from MEDLINE, 234 articles from PsychINFO, and 63 articles from SocINDEX were used for further analysis, totaling 716 articles. Using the Preferred Reporting Items for Systematic Reviews and Meta-Analyses (PRISMA) model as guidance (Figure 1), practical screening removed 170 duplicate articles and excluded 421 articles based on applying inclusion and exclusion criteria to abstracts. Full-text examination of 125 articles further reduced the final number of articles to be included in the critical appraisal for this integrative literature review. A final sample of 28 articles was selected for data extraction and critical appraisal (Table 1). The search terms and filters applied for each database are presented in Figure 2.

**Figure 3.**
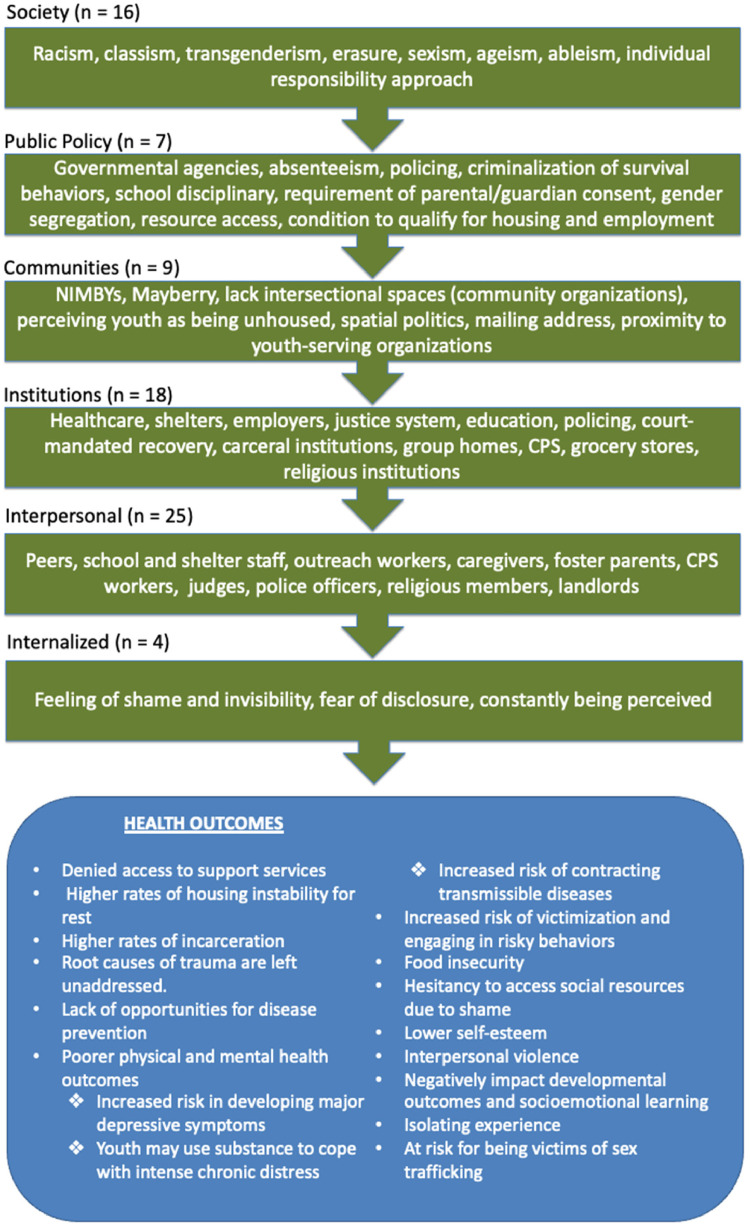
Socio-Ecological model of stigma and discrimination origins, and how they impact youth's health outcomes.

**Table 1.** Summary of Data Extracted for Integrative Review of Literature on Stigma and Discrimination of Unhoused Youth.

Author(s), date, location	Study design Sample size	Purpose	Stigma source	Discrimination source	Health outcomes	Implications	Critical appraisal score (Crowe)
Ausikaitis et al. (2015) Urban Chicago	Qualitative *N *= 18	To study experiences of unhoused youth in school and impact of McK-V Act	Peers, school staff	School administrators	Safety and hygiene needs not met between time spent outside of school and coming to school	Raise awareness of McK-V Act Remove penalties for tardiness and provide support to complete schoolwork. Have a “credit recovery program”	32
Barman-Adhikari et al. (2019) Denver	Qualitative *N *= 7	To explore unhoused youth's perspectives on current issues most important to them	Society	Society	Basic needs are not met consistently	Empowers youth to participate in policymaking, research, and program planning with rights-based approach Policies that address structural issues: basic income program, education access, and Housing First	31
Begun et al. (2020) Denver	Qualitative *N *= 30	To gather perspectives of unhoused youth on abortion	Society	Family, sexual partner	Interpersonal violence, harms from self-inducing abortions	Centers reproductive rights among youth Free, accessible, and contextualized education on healthy relationships	37
Brothers et al. (2020) San Francisco	Ethnography and qualitative *N *= 39	To examine relationship between PSH and food security	People, views around accessing food resources	Higher-end grocery stores, housing policy, housing residents	Food insecurity due to spatial food isolation, lack of affordable grocery stores, and discrimination Stigma causing people to AVOID utilizing free food sources Emotional toll of navigating harmful kitchen space with other residents	Secure funding for access to food Food security should be established upon first day of move in to PSH; accessibility in kitchen for youth with disabilities Protecting minoritized youth in communal spaces	32
Bruce et al. (2014) Chicago	Quantitative *N *= 200	To construct exploratory path models in describing relationships between minority stress, experiencing homelessness, and negative health outcomes	Home	Not discussed	Racial differences from impact of stigma Direct pathways were found between sexual orientation stigma and experiencing homeless Stigma internalization increases 2.5× risk of major depression	Assess parental acceptance of sexual orientation Peer-based programming	33
Crumé et al. (2019) Washing state	Secondary analysis *N *= 27,087	To study how housing instability and parental care instability impact youth's psychosocial development and function	Perceived stigma to disclose status with school personnel	School	American Indian/Alaskan Native, Black, and Latino students reported lower economic stability Lesbian and gay youth represented 3× in both housing and parental care instability	School should be positive, proactive, and observant to connect youth to social support services and prosocial activities Increase knowledge surrounding McK-V Act among families	29
Dang et al. (2019) Northen California Metropolitan	Mixed methods *N *= 22 ages 16–22, 68% male, 45% multi-racial (focus groups); N = 20, ages 18–24 interviews.	To utilize YPAR method in addressing STIs among youth who experience homelessness and finding effective factors in reducing STIs rate	Peers, internalized with fear of privacy on social media and communicating with sexual partner(s)	ED provider	Delayed care due to stigma and health insurance complexity Increased risk of receiving STIs due to lack of opportunities to discuss prevention strategies	Accessible community clinics with knowledgeable providers with drop-in sexual health testing services with free reproductive resources	39
Ensign and Panke (2002) Seattle	Qualitative *N* = 20	To examine experiences of unhoused adolescent women on reproductive health care	Providers	Providers, clinic staff	Delayed care Barriers to care: complex consent for care, address, ID, payment method, transportation, finance at clinic not specifically designed for unhoused youth	Nurse consulting line Allow youth to have a friend with them Including youth input in policy development	27
Ferguson et al. (2012) Los Angeles, Austin, Denver, New Orleans, St. Louis	Mixed methods *N* = 238	To study unemployment factors among unhoused youth	Not discussed	Employers, housing	Without employment, youth may be at higher risk of longer time spent being unhoused, engaging in survival behaviors, and misusing substance	Transform survival skills to job skills Use Social Enterprise Intervention and Individual Placement & Support models	35
Forge et al. (2018) Atlanta	Qualitative *N* = 4	To understand unhoused queer youth of color's lived experiences	Society	Family, friends, religious members, faith-based organizations, society	Risk for victimization and exposure to trauma due to lack of access to safe housing Food insecurity Risk for worsen mental health due to lack of support	Avoid giving youth “homelessness” label Use client-centered approach Connect sexual orientation and spirituality without shaming and in a trauma-informed way	30
Gattis and Larson (2016) Milwaukee	Quantitative *N* = 89	To study how stigma and discrimination associated with sexual minority, racial minority, and homelessness status impact youth's mental health outcomes	Not discussed	Healthcare, employment, education, justice system, law enforcement, housing	Depressive symptom and suicidality are correlated with stigmatizing and discriminatory experiences based on homelessness and racial minority status	Housing First and anti-racist policies Address discriminatory experiences by Black youth Research on prevention of initiation of discriminatory practices	29
Gwadz et al. (2009) New York City	Mixed methods N = 80	To study factors influencing the introduction of unhoused youth to the street economy	Interpersonal shame Perceived negative judgements Societal stigma	Employers, governmental agencies, banks, and customer services	Many have clinically significant depressive symptoms and substance use disorders Male-to-female transgender youth have high rates of discrimination and stigma Exposure to high health risks associated with street economy	Mental health care and career services to prevent recruitment into street economy Provide employers with incentives to hire youth with history of criminal activity Create more services that are LGBTQ + inclusive and not just “gay positive”	30
Harter et al. (2005) Ohio	Qualitative N = 48 youth + 12 administrators/staff	To examine processes contributing to invisibility of unhoused youth	People, peers, public policy, society	People, employers, community	Stigmatization can cause invisibility among youth to avoid further being stigmatized, reducing opportunities to access services	Disrupting discourses on Mayberry and NIMBY Recognizing street smarts as strengths and including teaching methods that encourage meaningful dialogue, truth sharing, cross-interactions, and draft of new narratives	27
Hickler and Auerswald (2009) San Francisco	Mixed methods *N* = 54 (ethnography) =205 (quantitative)	To compare experiences of entry to homelessness, self-perception, and survival strategies among white and African American unhoused youth and their variations on health behaviors and health outcomes	One racial group of unhoused youth judging the other racial group Survival activities being mocked Shelters and labels	Racist shelter residents Governmental agencies	Recently incarcerated youth who became unhoused were described as incredibly vulnerable White unhoused youth reported significantly higher rates of injection drug use and of hepatitis C than African American unhoused youth	Youth shouldn't be asked to identify themselves as unhoused to access support. Providing housing for youth involved with foster care and carceral system	30
Holger-Ambrose et al. (2013) Minneapolis	Qualitative *N* = 13	To gather perspectives of unhoused youth who have been sexually exploited on how street outreach workers can improve outreach and coordinate service connection efforts.	Outreach workers, female-identifying individuals who don't engage in sex work	Not discussed	Interpersonal violence, coercion into engaging sexual activities and using substance, isolation	Incorporate best practices for outreach to youth who engage in sex work Policies: creating pathways within juvenile justice, foster care, and mental health care to prevent and help exit sexual violence and/or homelessness	30
Hudson et al. (2010) Santa Monica	Qualitative *N* = 24	To understand unhoused youth's barriers and facilitators to access services and how youth would like to improve on current social programs	Law enforcement, judges, passerby	Interpersonal, healthcare providers, police, policy	Chronic health conditions aren't being properly managed due to barriers to obtain governmental support lack of interpersonal support and encouragement	Providing outreach for mental health care, with flexible clinic hours and expand services to decrease wait times	35
Semanchin Jones, Bowen & Ball (2018) Buffalo, NY	Qualitative Descriptive *N *= 20 Age: M = 19.9 (SD = 1.65) yrs. 10 African Amer 3 White 7 Mixed or other 6 Latinx Ethnicity	Explore lived experience of youth involved with child welfare and homeless serving systems; and challenges with educational system.	Legal guardians Peers	Service providers, school staff, teachers, CPS workers, educational system, school disciplinary policies (e.g., parental/guardian consent excludes youth from normalizing activities.	Continuous abuse at home, unaddressed root causes of traumatic experiences.	Review of school policies and staff training.	37
Milburn et al. (2006) Los Angeles	Quantitative *N* = 227	To study discrimination experiences among unhoused youth	Not discussed	Peers, police, family	Unhoused LGB adolescents experienced significantly higher rates of discrimination than heterosexual adolescents (81% v. 59%)	Additional research on discriminatory experiences among LGB homeless youth consider family influences on adolescent's experiences	36
Moore et al. (2019) California	Longitudinal *N *= 390,028 50.9% female, 23.7% White, 48.6% Hispanic, 3.6% Black 10.4% Asian; 3% AI,AN, HI, PI; mixed 10.8%	Compare violence and safety experiences of students who experienced homelessness and those who did not.	Not discussed	Discriminatory bullying, behavioral victimization, and weapon victimization at school.	Affected developmental outcomes and socioemotional learning	Need for collaboration between community, school, housing, and healthcare. Assess students’ exposure to violence. Apply ESSA and Title IX to prevent discrimination	32
Moore et al. (2020) California	Quantitative *N *= 389,569	To examine how violence and climate at school can influence support for unhoused students	Not discussed	Peers	Violence including behavioral victimization, discriminatory bullying, and weapon involvement	Establish data collection guidelines to track school's needs in serving unhoused students Promote school safety and sense of connectedness	34
Reck (2009) San Francisco	Qualitative *N *= 5	To explore experiences of LGBTQ youth of color experiencing homelessness	Castro residents, community	Housing renters Residents nearby youth organization Public spaces beyond Castro Castro residents Police	Experiencing higher risk of victimization, harassment, violence, and marginalization based on age, race, socioeconomic status, and gender	Recognize intersectionality in building inclusive spaces	22
Robinson (2018) Central Texas	Qualitative *N *= 40	To examine how LGBTQ youth who experience homelessness perceive role of child welfare system on their housing status	Emergency shelter, non-intersectional spaces	CPS, shelter, residential treatment centers, psychiatric hospitals, foster families	Denial of access to transgender care, increased social isolation Target of violence on basis of race and sexual orientation Worse quality of life from forced institutionalization Limiting permanency	Implementation of gender-affirming policies Re-examine policy of having foster parents notifying DFPS for placing youth in psychiatric hospitalization	29
Robinson (2020) Austin and San Antonio	Qualitative *N *= 40	To document how LGBTQ youth experiencing homelessness are regulated by police and state agents on gender identity and expressions, and sexual activities	Police	Police, court-mandated recovery programs and their staff, individuals within prisons, staff of LGBTQ + shelter	Youth suffering from violence and decreased safety because policing practices marginalizes them instead of uplifting and protecting them	Research: ethnography on manifestations of trans-profiling, racism, and classism through policing; how gentrification by White middle class individuals further marginalize LGBTQ youth of color who are poor	33
Santa Maria et al. (2015) Houston, TX	Mixed methods *N *= 64	To study the effects of being unhoused on sexual risk behaviors and victimization on youth	People, internalized	Not discussed	Stigma increased risk for involvement in risky sexual behaviors, contracting STIs, and higher risk of substance use Victimization at early age with highly offensive and stressful circumstances	Assess housing status Support transition time into early adulthood Research on relationship between stress and risky sexual behaviors, along with impact of stigma and self-reliance	38
Schmitz and Woodell (2018) Nebraska	Qualitative *N *= 22	To study the influence of religion on LGBTQ unhoused youth's experiences	Religious institutions, relatives	Religious institutions, relatives	Religious beliefs and practices can be both rejecting and used as coping mechanisms at the same time.	Promote relational health Utilize intersectionality in developing interventions	34
Schmitz and Woodell (2018) Nebraska	Qualitative *N *= 46	To explore how unhoused LGBTQ youth who is enrolled in school cope with familial reactions to youth's gender and sexual identity	Family	Family, peers	Lower self-esteem Detachments from social networks and social services	Consider challenges navigating socioeconomic environment and family relationships among LGBTQ + youth	32
Shelton (2015) New York City	Qualitative *N *= 27	To study the experiences of transgender and gender expansive youth who are unhoused	Transgender individuals are viewed to have a certain look, LGB serving organizations, school	Shelter policies, shelter residents, shelter staff, workplace, societal erasure, school, home	Misgendering and lack of inclusive intake questionnaires can cause anxiety, isolation, and harm associated with services Lower self-esteem and higher risk of depression from victimization	Training on harms of cisgenderism and inclusivity toward transgender and gender expansive youth Allow more than 60 days to obtain IDs, particularly for emergency shelter and transitional living programs Policy focus and shelter beds for youth age 21+	26
Shelton (2016) New York City	Qualitative *N *= 27	To study how transgender and gender-expansive youth conceptualizes homelessness	Family	Family, group home, structures	Intense distress, depression, isolation, violence from living on the streets and pressure to conform	Assess housing context and provide spaces for sexual and gender identity development Policy: include nondiscrimination protections in RHYA Organizations: facilitate making permanent connections with adults Research: Evaluate programs and local policies impact on transgender and gender-expansive youth	30
Toolis and Hammack (2015) Chicago and Seattle	Qualitative *N *= 11	To explore how unhoused youth create meaningful lived experiences and process stigmatizing ideologies against them	Interpersonal, Society	Institutions/service organizations, school officials and police, shelter staff, classmates	Social isolation, domestic violence, may not be able to seek safety or protections from authorities when needed, lack of basic needs met	Inquire about and trust the knowledge that unhoused youth possess. Engage youth in making structural changes	32

CPS = child protective services; DFPS = Department of Family and Protective Services; ED = emergency department; ESSA = Every Student Succeeds Act; LGBTQ + = Lesbian, Gay, Bisexual, Transgender, and Queer; IPV = interpersonal violence; McK-V: McKinney-Vento; NIMBY = not-in-my-backyard; PSH = permanent supportive housing; RHYA = Runaway and Homeless Youth Act; YPAR = Youth Participatory Action Research; PSH = permanent supportive housing.

### Critical Appraisal

The Crowe Critical Appraisal Tool (CAT) was used to determine the quality of research studies with a potential 40-points format. The Crowe CAT asks questions about quality in eight sections: preamble, introduction, design, sampling, data collection, ethical matters, results, and discussion (Crowe et al., 2012). The preamble includes details about the title, abstract, tables, and figures. Each of the eight sections can be scored from 0 to 5 points, with the total of 40 potential points. This CAT has evidence of validity and reliability and has the versatility of being applicable to any research design, including those that are qualitative and quantitative (Crowe et al., 2012).

### Data Extraction Process

The 28 peer-reviewed articles selected were analyzed by both authors to extract information on article authors, date, location, study design, sample size, purpose, sources of stigma and discrimination, health outcomes, implications, and CAT scores. Both authors met regularly to compare findings, reconcile disagreements, and reach consensus on data extracted. Data is presented in Table 1.

## Results

A total of 808,296 participants were in the final sample of this review. Participants lived across the United States in 13 states: California, Colorado, Georgia, Illinois, Louisiana, Minnesota, Missouri, Nebraska, New York, Ohio, Texas, Washington, and Wisconsin. All studies included a discussion on how unhoused youth experienced social stigma and/or discrimination. Table 1 is included to show how unhoused youth experience social stigma and/or discrimination. Categorization through the application of the socio-ecological model resulted in 16 studies categorized at the societal level, seven studies at the public policy level, nine studies at the community level, 18 studies at the institutional level, 25 studies at the interpersonal level, and 4 studies at the internalized/personal level. Most of the studies were qualitative (*n *= 18), with almost equal numbers of studies that were quantitative (*n *= 6) and mixed methods (*n *= 5).

Researchers discussing stigma sources (*n *= 24) highlighted shame around accessing social services, judgments from peers and school staff, lack of intersectional spaces, looking down on survival strategies, and additional stereotypes around race, ethnicity, LGBTQ+ status, and disability. Healthcare (*n *= 2), school (*n *= 2), shelters and service-providing organizations (*n *= 4), policing (*n *= 3), and public policy (*n *= 1) were discussed on misconceptions around unhoused youth. Harter et al. (2005) reported how Mayberry Not-In-My-Backyard (NIMBY) public policies tended to limit youth-serving organizations from existing and categorized unhoused youth behaviors as unsafe.

Studies discussing discrimination sources (*n *= 26) highlighted treating unhoused youth with harsh requirements, degrading a youth's ability to care for themselves, excluding services due to criminal history background, perpetuating racism and transgenderism, and criminalizing survival behaviors. Healthcare (*n *= 4), school (*n *= 5), shelters and service-providing organizations (*n *= 10), policing (*n *= 5), and public policy (*n *= 1) were identified as potential sources for the constant violence against unhoused youth at services supposedly intended to help them survive, thrive, and exit homelessness.

Crowe scores of the papers in this review had a median of 32 points, mean of 31.75 points, and a range from 22 to 39 points. Both authors of this review read all papers in the final sample and discussed differences in their assessments to reach consensus on the final scores. The majority of papers in this review scored above the mean, which meant that nearly 80% (31.75/40) of the items on the Crowe CAT were answered affirmatively. For those studies that scored below the mean and toward the bottom of the range (e.g., 22/40 = 42.5%) were less trustworthy. Those studies had a higher risk of bias than those with higher scores.

### Health Outcomes

*Housing instability.* Many studies reported increased housing instability due to stigma and discrimination (Table 1). For example, Robinson (2018) stated that gender segregation housing and denial of correct gender housing placements can lead to increased evictions and limited permanency. Within shelters, forms of violence such as harassment and victimization can be rooted in minoritized identities against unhoused youth, especially by shelter staff and other residents. Through stricter requirements and enforced by policing, YEH are under more frequent surveillance, leading to an increased risk of incarceration (Robinson, 2018).

*School violence*. Strong protections against unequal treatment for unhoused youth are needed to prevent trauma and marginalization. Moore et al. (2020) highlighted that stigma and discrimination within school can negatively impact socioemotional learning and appropriate developmental outcomes. With fewer opportunities to build strong social networks and receive encouragements, unhoused youth are less likely to have access to a safe space for optimal learning (Crumé et al., 2019). Unhoused youth who encounter these harmful interactions every day at school are at risk of not being able to complete their education and have a higher chance of dropping out to avoid further trauma (Ausikaitis et al., 2015). School should be conducive for learning and socialization, where youth feel comfortable and trust the institution to support them.

*Denied access to care.* Healthcare providers may create a hostile environment where unhoused youth do not feel welcome in receiving care, as documented in Table 1. Ensign and Panke (2002) and Dang et al. (2019) stated that judgment, lower quality of care, and providers assuming unhoused youth as being ignorant about their health have contributed to limited discussion of prevention strategies. They also found that unhoused youth of minoritized identities experience other forms of oppression from providers and clinic staff, with little regard to privacy and confidentiality, misgendering, and stereotypes associated with racism, classism, sexism, ageism, and ableism, showing consistency with intersectionality theory.

*Worse health outcomes*. Numerous studies discussed internalization of stigma and pervasive discrimination on youth well-being (Table 1). Increased risk of contracting transmittable diseases (such as sexually transmitted diseases), developing mental illnesses from chronic stress, major depressive symptoms, suicidality, using substances to cope, food insecurity, exposure to violence, being victims of violence, and lower quality of life and opportunities in managing chronic diseases were commonly found health outcomes that are consequences when unhoused youth are not recognized as humans deserving equitable treatments and support services (Ausikatitis et al., 2015; Brothers et al., 2020; Bruce et al., 2014; Crumé et al., 2019; Dang et al., 2019; Gattis & Larson, 2016; Hudson et al., 2010; Reck, 2009; Santa Maria et al., 2015; Semanchin Jones, Bowen, & Ball, 2018; Shelton, 2015; Shelton, 2016; Toolis & Hammack, 2015). These health disparities further marginalize the population from thriving.

## Discussion

It is evident that unhoused youth experience interconnected social stigma and discrimination. Numerous aspects of youth's lives, such as accessing education, healthcare, and community spaces, are often highly regulated on how youth should present themselves. Using the socio-ecological model and intersectionality framework, unhoused youth have shown to be victims of classism amplified by other forms of identity-based oppressions, such as racism, sexism, transphobia, ableism, etc. NIMBY and Mayberry public attitudes and policies contribute to the call for increased policing and surveillance. NIMBY was defined as opposition to development of housing and other community facilities, usually rooted in the assumption that current residents’ home values and quality of life would decrease (Pendall, 1999). Mayberry, on the other hand, denoted the lack of awareness of community issues, such as youth homelessness, and highlighted the need for order and utopia (Harter et al., 2005). Without an acknowledgment that youth homelessness exists and that social community resources such as supportive permanent housing are needed, unhoused youth face immense challenges in exiting homelessness.

It is concerning that spaces intended to be supporting unhoused youth toward journey of being permanently housed safely can become extremely harmful as to limiting access. In mapping out a nonexhaustive list of how, where, and who drive stigma and discrimination (Figure 3), this integrative literature review offers a starting point to eliminate barriers for unhoused youth.

### Implications

*Nursing.* Nurses work in inpatient and outpatient settings with a holistic view of the patients, as well as nonclinical settings. Nurses should ask for inclusive healthcare delivery training that includes continuously reflecting on their own assumptions, involving youth in decision-making, knowing how to assess social needs, and understanding how health disparities are perpetuated for unhoused youth. Nursing as a profession can hold an individualized and systems-level critical view towards the patients; understanding that systems drive certain health behaviors that produce certain health outcomes, and currently, systems supporting unhoused youth do not allow space for them to make decisions that achieve economic mobility and facilitate their exit from homelessness. Nor do these systems help to prevent re-entry into homelessness. Nurses are called upon to use a trauma-informed care model to acknowledge youth experiences and establish trust within therapeutic relationships. Nurses can become engaging health educators in making prevention health information more accessible to youth and how they can be connected to resources for applying this knowledge. Nurses need to be at the forefront to advocate for antiracist, gender-affirming, and inclusive built-care environments to address compounding the effects of stigma and discrimination through the lens of intersectionality. Public health nurses have skills to collaborate with street outreach workers and address health and social issues at the same time.

*School.* Schools are encouraged to implement evidence-based strategies, especially because schools are often the primary and only place in which unhoused students can access care. School nurses can be advocates and leaders in developing and directing programs and practices for this vulnerable population. Throughout stages of engagement, unhoused youth need always to be included in planning and decision-making, such as serving on advisory boards. Additionally, school nurses can utilize Every Student Succeed Act (ESSA) to request more funding for supporting unhoused youth through Titles I, II, and IV for activities, including school-wide housing needs assessment, providing trauma-informed care, creating antidiscriminatory learning environment, supplying items to fulfill basic needs (clothing, hygiene, food, sleep, and social network), coordinating with local partners in supplying housing, and advocating for housing to be included as a measurable indicator for academic success (Blackborow et al., 2017; Moore et al., 2019). School nurses can further request funding for the professional development of staff, particularly in areas of applying structural competency and intersectionality to view homelessness as a solvable structural problem (Blackborow et al., 2018). Some other interventions that schools can implement:

1. Use Positive Youth Development Framework, such as providing opportunities for group and individual recreational activities (Benson et al., 2006).
2. Implement alternative pedagogy that engages traditional knowledge and lived experiences.
3. Provide opportunities for healthy identity exploration and relationship building among classmates and school staff.
4. Advertise the role of McKinney-Vento Act liaison to increase outreach to youth living in unstable housing (National Center for Homeless Education, 2018).
5. Proactively screen students and households for risk of homelessness, as well as social needs.
6. Compile local resources for unhoused youth and support in navigating those resources
7. Transition towards a model of compassion and support in understanding causes of why students may miss school or changes in behaviors, and away from the model of punishment and criminalization.
8. Believe in youth when they choose to disclose about housing status and other health concerns.

*Policy*. Policy changes are cornerstones for producing sustainable impacts, especially at the structural level. Education systems can pursue the following policy recommendations that support school nurses’ efforts to address social stigma and discrimination against unhoused students and youth at large.

1. Investing in schools to ensure that they are staffed with nurses, particularly for schools in under-resourced communities
2. Advocating for expansion and protection of youth's human rights in areas such as housing, anti-racism, reproductive health, LGBTQ+, disability, etc.
3. Investing in community organizations, clinics, building intersectional spaces, and programming activities that foster positive relational health and keep youth safe.
4. Expanding policies that promote economic mobility, such as tax credit, raising minimum wage, basic income program, access to affordable healthcare, affordable education, and job training programs, etc.
  Unhoused youths’ needs span beyond educational spaces. Additional policy recommendations are discussed in addressing youth homelessness, stigma, and discrimination that require larger societal responses. The Housing First model has proved its powerful effectiveness in improving health outcomes, reducing visits to emergency rooms, and increasing housing stability long term (Baxter et al., 2019). Housing First model is guided by the core principles of: Everyone has the right to housing, little to zero conditions in accessing housing, using support services is voluntary, and rapid entry to housing (Baxter et al., 2019). Therefore, Housing First should be considered as a standard in addressing homelessness on organizational and public policy levels. Social services can then be provided as wrap-around, with basic needs being prioritized at the same time as housing placement. For non-HUD-funded social housing, policies containing language like the Equal Access Rule should be adopted, especially in state, city, and privately funded housing. Shelters and other forms of housing need to re-examine cisgender policies to be more inclusive of transgender individuals. Unhoused youth who are 21 years of age and older should also be considered a particular focus for public policy support, as they face immense challenges navigating young adulthood, a demanding workforce, and independent living.
  Healthcare systems have an opportunity to re-examine their triage and discharge policy guidelines for unhoused youth, collaborate with community partners, and train their staff on structural processes impacting re-admission for unhoused youth. The evidence of stigma and discrimination against unhoused youth within healthcare is enormous. Therefore, healthcare systems and their clinicians need to incorporate access to housing as part of the discharge process, effective triage processes that do not exclude people who experience homelessness and instead prioritize objective health needs, and employ health system navigators who are contextually aware and proficient in cross-system communications. Clinical nurses are often the first members of the care team who unhoused youth encounter in hospital settings. Therefore, nurses are a powerful force in shaping positive experiences of accessing healthcare.
  Some other include nondiscrimination clauses and an enforcement mechanism into Runaway and Homeless Youth Act (Family and Youth Services Bureau, 2018)
5. Hold law enforcement accountable for discrimination based on profiling and unjust civilian violence.
6. Adopt policy to ensure adequate affordable housing and limit gentrification that displaces people.
7. Create safeguards for successful transition out of foster care, justice system, and mental health care that prevent (re)entry into homelessness and victimization.
8. Expand technology and broadband access.
9. Remove criminalization of survival strategies.
10. Provide incentives for employers to hire youth with a criminal history.

### Limitations

Despite the extensive nature of this review, several limitations are important to address. Studies included are written in English and conducted within the United States. Therefore, the experiences of unhoused youth may not be fully captured in other contexts. The decision to exclude op-eds, case reports, and commentaries may have also limited the extent of stigma and discrimination against unhoused youth. However, this creates an opportunity to acknowledge that this issue spans more than personal responsibility; it calls for a system approach, policy-driven, and greater involvement of nurses in ensuring the excellent health of unhoused youth. The Crowe Appraisal tool asks users to provide a subjective score after considering reflective questions. It can create disparity in scoring article quality. Four of the included studies scored below 28 points (below 1 standard deviation to the median of the critical appraisal score), with 24 studies scoring above 28 points. Future research studies should also explore how stigma and discrimination are perpetuated within the research realm, the effectiveness of housing-related programs, and how to drive culture around addressing homelessness away from criminalization and towards strong social commitments. Additionally, the article selection process was completed in April of 2021, which does not contain any new studies published after that time. Since then, more studies may have also addressed the issue of social stigma and discrimination against unhoused youth. Future research studies should examine articles after April 2021.

## Conclusion

Unhoused youth experience multi-level challenges to survive and balance a dynamic period in their lives. Social stigma and discrimination complicate those challenges even further, limiting youth from opportunities that can ensure their wellness and positive developmental outcomes. Every nurse within schools and the community needs to be committed to creating a welcoming environment for unhoused youth, working in partnership with them in addressing their needs, and advocating for a just system through policy changes that prioritize unhoused youth's perspectives, health, and access to housing.
